# Laboratory Findings as an Aid to Diagnoses

**Published:** 1916-08

**Authors:** Nannie Hamilton

**Affiliations:** Birmingham, Ala.


### LABORATORY FINDINGS AS AN AID TO DIAGNOSES

By Miss Nannie Hamilton, Birmingham, Ala.

Some one has said, "a common characteristic of the human family is the enthusiasm with which they ride their hobbies," and so it is with pleasure that I speak to you today on Laboratory work, notwithstanding the fact that when one of so small knowledge, addresses so learned a body, she places herself in the same unenviable position as the actors in the classic little poem called "Watchful Waiting," which reads something like this:

"The cat was in the pantry
a layin' for a rat.
The boy was 'round the corner
a layin' for the cat.

The cat was named Jemime
The boy was named Jim,
And his father's upstairs, just
a layin' for him!"

But as I come, not in the attitude of instructor, to tell you of things you know not of—but only to call to your attention a few points which in your larger work you sometimes forget, I beg you to be lenient.

A clean cut diagnosis, needs no illuminating sidelights, there is nothing truer than this, unless it be, that a diagnosis which is not clear-cut, does! and the laboratory should furnish one of the lights.

I shall invite your thoughts for a short time, to a few of the questions which engage the attention of the laboratory worker: Blood examinations, Vaccines, Wassermanns.

Under the subject of Blood let us glance for a moment at the white cell count, and see what an important role it plays, as an aid to diagnosis. Whereas a hyperleukocytosis most often depends upon an increase in the polynucler neutrophiles it is not always so, and there have been times when the surgeon's reputation was saved by a careful examination of a blood sliding, for scarcely second in importance to the total count is the differential count, which is so often neglected. Simon lays such special emphasis upon it, even when the septic condition is recognized. An important point to be remembered in this condition is, that a drop in the total number of leukocytes, does not always mean improvement. A decrease in the total count with a rise in the per cent. of "polys" occurs as soon as peritonitis intervenes, even before it is apparent in other ways, that the patient is in a more serious condition, and unless a differential was made at the same time as the total count, a change later in the relative percentage of cells could not be appreciated.

Another point which is sometimes not considered, is the fact that the normal percentage of the cells in childrens' blood differs from the normal adult blood, in inverse proportion to the age of the child up to 10 years. And not only in sepsis is a differential of importance. If one is familiar with the varied blood findings of the different diseases, a count of 500 cells will give you a correct blood picture of the case in point, and often the suspected diagnosis is confirmed or excluded by elimination. To illustrate.

Example 1. A young woman came to a specialist to have her throat examined. He found her tonsils large and protruding, and at once advised that they be removed. The operation was set for next day. As she was leaving he noticed her extreme pallor and she was sent to the laboratory for a Hg. estimation. It was seen that the blood was thin and somewhat milky looking, so several slides were made and stained. Upon a glance at these, total counts were also taken and a differential made. The complete report read as follows: Hg (Sahli) 30 per cent., Red Cells 3,000,000, White Cells 250,000. The differential showed the increase due to the lymphocytes, and that the relative percentage of these cells to other cells was 90. Thus the blood picture of Lymphatic Leukemia was presented, and the operation postponed.

Example 2. Mr. F. entered the hospital in an extremely ill condition. Very little previous history could be obtained except that he had been sick a long time. Upon examination heart was found hypertrophied, abdomen tympanitic. There was slight pulmonary eodema and high blood pressure. The liver was enlarged and of irregular outline, and a distinct tumor felt which was thought to be a liver abscess. A complete blood picture was asked for together with a sputum and urine ex. The report read as follows:

W. B. C. 11,000.

Differential, L. 41—M. 9-P. 44—E. 6.

Hg. 47 per cent.

Red Cells 3,280,000.

Widal and Malaria negative and sputum neg. to T. B. The urine showed a spg. 1005, also alb. few white and few red cells, casts of all kinds.

Some time later in the day the resident physician made a total count and reported 15,000 leukocytes, and upon this count the surgeon operated. It is evident that the laboratory findings were discredited. The patient was put to sleep under gas, and abdomen opened to insert a drain in the suspected abscess, but no abscess was found. The patient was returned to his room, where he died a few hours afterwards. On autopsy, sections of liver and kidney were obtained, and upon cutting and staining the same, a text book picture was seen of a syphiletic liver, and of a chronic intestinal nephretis,—which bore out laboratory findings.

Case 3. And another example. A baby of about two years old, developed high fever without a cause that was apparent. A Widal and smear for malaria were sent to laboratory. Both were negative, but upon a differential being made, it was found the percentage of L—26, M. 7, P. 67, with an absence of E's. This was reported to the doctor who said "much obliged, but you have not helped me!" Upon which the laboratory replied: "but we have, for the differential shows the "polys" to be 20 per cent. above the normal for a child his age, and this together with an absence of eo-sinophiles suggests an inflammatory or infectious process. The next day there was a well defined pneumonia.

Case 5. Mrs. A., an aged woman, was brought to a hospital "for operation." Upon examination a "mass was found in the abdomen, and the resident physician reported a leukocytis of 16,000. No other blood work was done. Fearing the anaesthetic on account of a "bad heart," an attending relative asked for "consultation;" when the medical man came he requested a Hy. estimation, and examination of a blood slide. The latter showed estivo-autumnal parasites in great numbers and the differential count of white cells a relative lymphocytisis. The correctness of the reported total count is questioned. Distinct peukopenia or at least an absence of leukocytosis is almost invariably found in malaria, and should in this case a septic condition have been present, the differential would not have shown a lymphocytosis, but a "poly-cytosis." In the light of these facts the mass in the abdomen was easily seen to be the spleen!

Case 5. Another example showing that an increased leukocyte count should be checked by a differential, occurred not many days ago, when a child was brought into hospital with temperature, and pain in abdomen. A leukocyte count was made and reported to be 13,000. The child was operated upon and a perfectly normal appendix was removed! Recovery from the operation does not seem to be interfering with this case of typhoid.

These few examples prove the importance of a careful blood examination taken beyond the making of a total count, and also hints that every one, can not make a white blood count, especially when the red blood pipette is used for same.

In any case which shows a leukocytosis, especially a mild leukocytosis, a differential should be employed to prove the suspected septic condition; and of normal values in the various percentages of cells be reported, then rest assured, there has been some slip of technique,—either in taking the blood, or in diluting the same. And another point,—only by a cell count of no less than 300 cells, preferably 500, can a correct blood picture be obtained.

Three diseases, especially of which we learn more by examination of the red cells, are pernicious anemia, secondard anemia, and chlorosis. There are many points of similarity, many points of difference between them which can not always be learned by one examination. Then too, so often only a partial examination is asked for, and on this partial examination will the physician hang his hopes of elucidating a problem.

For instance: a blood smear is sent in to the laboratory and the question asked: "What is it?" No knowledge of the red count, leukocyte count Hg. is given,—probably not known to the doctor himself, all three of which are most important in the differentiation required. Let us compare these blood pictures and see how little can be learned by the stained specimen alone.

First, Pernicious Anemia. Except in Ehrlick's "plastic anemia," a fatal type in which the bone marrow so utterly fails to compensate the blood loss, that erythroblasts of all kinds are wanting,—nucleated reds are always found, unless in periods of remission, and the diagnostic point is the predominance of the megloblastic type. In no other condition except Nitrobenzol poisoning, and the high-grade anemia caused by the infection of the Bothrocephalus latus, do we get this picture. But should we examine a slide during remission, with no knowledge of the Hg. total tumber of reds, or existing leukopenia nothing but a negative report could be rendered, and more than that, no hint as to the possibility of the disease. For a severe secondard anemia could give the same stained picture, and only the difference of a high or low color index, number of red cells, and percentage of Hg. together with a total and differentia leukocyte count, could enable one to express an opinion.

As for Chlorosis—there is no blood picture peculiar to this condition. Changes precisely similar are to be seen in secondary anemia especially those brought about by Syphilis, Septicemea, and chronic renal lesions. The low color index, the general decrease in size of cells, the normal number of leukocytes, associated with a relative lymphocytosis,—are only highly suggestive. The difference between chlorosis and pernicious anemia is more easily made, though not from the stained smear alone. Thus we see, under some circumstances, the laboratory is not at fault, when desired information is not forthcoming.

The isolation of an organism or organisms, causing a morbid process should be the first step, from a laboratory standpoint, in the beginning of Vaccine Theraphy. For in doing so the "missing link"—or the link that's often missed—is thus supplied between the patient and recovery.

When we hear the confusing views as to the efficiency or non-efficiency of vaccines, when we see such splendid results obtained by some, and such signal failures by others, we know there must be some explanation to this apparently unexplainable fact.

A few years ago, all vaccines were made from the patient's own lesion. Only men undertook this work whose previous training qualified them to perform the required bacteriological examination and having isolated beyond a doubt the specific organisms, it was re-introduced into the patient to stimulate the production of anti-bodies. The success attending these men soon claimed the attention of the laity, the medical men in general, and the manufacturers in particular. To meet the demand for cheapness and convenience, the commercial vaccines soon flooded the market, made from all probable (and possibly) improbable pathogenic bacteria. In using these vaccines when the infecting organism is in pure culture, the patient stands a better "chance" of cure than when different bacterial species produce the morbid condition. This of course was realized by the manufacturer, but with this disquieting thought,—came the wonderful inspiration of that gun shot prescription, called mixed vaccines.

Now if we keep in mind that an active resistance against a harmful parasite, brings about immunity, and as an outcome of this state of immunity the body possesses a variety of protective substances called antibodies, then we also see that if a mistake is made and a different harmful agent other than the one already at work be introduced, the immunity built up by the active resistance against this agent, does not produce any protectice substances or antibodies, to fight the original infection. We have heard of the physician who was called in to see a patient with an obscure malady. Knowing relief was expected of him by family and friends, he gave a powder which quickly produced fits. He could cure fits!

To obtain the desired results in the use of vaccines one must know four things besides the pathogenic organism: (1) Under what conditions to begin the treatment, (2) the knowledge of the proper dose; (3) the effects to be looked for (4) and when to repeat the dose.

The complement fixation test for syphilis has proven to be one of greatest value, not only as an aid to diagnosis, but as a control of treatment, and an index as to the efficiency of treatment.

Possibly no one test, that is being made by the laboratory today; so aids the clinician as this,—especially in obscure cases.

A good example of this came under observation a few months ago. A baby was brought to a doctor with a history of having been sick all of its life. After a careful examination nothing of any special interest could be found to explain the trouble. The child was put on some digestice treatment, and kept under observation for several months. At the end of this time he was no better. Finally a Wassermann was made. The report rendered was a 3 + reaction. Not until specific treatment was instituted did the child begin to gain.

Like many other laboratory tests, a positive Wassermann is of more significance than a negative one, especially as the routine examination now is usually done on the blood serum alone. Recognizing the frequency of early envolvement of the nervous system, due to attack by a special strain of Spirochetes, with a predilection for the nervous structures, it is advisable to make studies not only on the serum, but on the spinal fluid as well, especially when a "negative" Wassermann is at variance with clinical signs. Cassonte has recorded three cases of dementia precox development at the age of 30 years,—"like a bolt out of a clear sky"—with nothing in the patient's past to indicate syphilis, except a history of a "nervous child." Had the spinal fluid been examined and treatment begun, this ir-rer arable injury to the nervous system might have been warded off.

Another reason why a positive Wassermann is of more value than a negative one, is shown by the work of Craig of the U. S. Medical Corps, who by repeated titrations on sera of untreated cases, proved the variability, from day to day, that occurs in the complement binding power of these sera, and shows that this natural variation must be likewise reckoned with, while the patients are under treatment.

It is evident from this that a single negative Wassermann is not proof positive of the elimination of the disease.

In doing a number of Wasserman fixation tests in conjunction with the luetin test, it is interesting to see how far from parallel they run. This is not strange, however, when we remember that the reaction of the leutin is due to the true pallada antibodies, which persists for a longer period of time, than the pallada toxins. These toxins it is believed give rise to the reactionary products of the body cells, which are responsible for the Wassermann.

Therefore a repeated negative Wassermann of both blood and spinal fluid, with a positive leutin, does not necessarily indicate that further treatment is required. And again a negative Wassermann, using an antigen reinforced with cholestesrim, is stronger evidence of the absence of syphilis, than is a negative leutin. For Noguchi has shown that the luetin is generally negative in the primary and secondary, untreated stages of syphilis and that its principal value is in latent and tertiary syphilis and to show whether a patient with a negative Wasermann has ever been infected.

I have attempted to show you how the laboratory, in many ways, could be of more value to you, and the importance of co-operation.

To the practitioner who is well versed in laboratory work, who appreciates the importance of its findings, who also appreciates its limitations; to the man who has in his mind's eye a clear blood picture of the various diseases, a bare report of the examination is all that is necessary to him. But one whose many duties prevent his keeping up along these lines, is more dependent upon the laboratory for the interpretation of its findings, and for the worker to assume this added responsibility, she must be given such "facts as will allow the most intelligent study of the specimens before her."

Just as the best results cannot be obtained between anaesthetist and surgeon without "team work," so in many instances the most efficient service capable of being rendered by the laboratory is defeated by a failure on the part of the physicians to send with the specimen some history of the case.

N. B. Hamilton.

---

### DO YOU KNOW THAT—

Sags in roof-gutters may act as mosquito breeding places?

America's most valuable crop is babies?

The public cigar-cutter is a health menace?

The United States Public Health Service maintains a loan library of sterioptico slides?

The typhoid rate measures accurately community intelligence?

Whooping cough annually kills over ten thousand Americans?

Bad housing produces bad health?

Rocky Mountain spotted fever is spread by a wood-tick?

